# Machine Learning-Based Damage Diagnosis in Floating Wind Turbines Using Vibration Signals: A Lab-Scale Study Under Different Wind Speeds and Directions

**DOI:** 10.3390/s25041170

**Published:** 2025-02-14

**Authors:** John S. Korolis, Dimitrios M. Bourdalos, John S. Sakellariou

**Affiliations:** Stochastic Mechanical Systems & Automation (SMSA) Laboratory, Department of Mechanical Engineering and Aeronautics, University of Patras, 26504 Patras, Greece; i.korolis@ac.upatras.gr (J.S.K.); dimitrios.bourdalos@ac.upatras.gr (D.M.B.)

**Keywords:** damage diagnosis, floating wind turbine, varying wind conditions, vibration signals, machine learning methods

## Abstract

Floating wind turbines (FWTs) operate in offshore environments under harsh and varying operating conditions, making frequent in situ monitoring dangerous for maintenance teams and costly for operators. Remote and automated diagnosis, including the stages of detection, identification, and severity characterization of early stage damages in FWTs through advanced vibration-based structural health monitoring (SHM) methods of the machine learning (ML) type, is evidently critical for timely repairs, extending their operational lifecycle, reducing maintenance costs, and enhancing safety. This study investigates, for the first time, the complete (all stages) damage diagnosis problem by employing well-established ML SHM methods and conducting hundreds of experiments on a lab-scale FWT model operating under different wind speeds and directions, both in healthy and damaged states. The latter include two distinct blade cracks of limited length, two added masses attached to the blade edge simulating possible accumulation of ice, and connection degradation at the mounting of the main tower with the floater. The results indicate that the proper training of advanced ML methods using damage-sensitive feature vectors that represent the structural dynamics within the entire frequency bandwidth of measurements may achieve flawless damage diagnosis, reaching 100% success at all diagnosis stages, even when only a minimal number of vibration signals from a limited number of sensors (a single sensor in this study) are used.

## 1. Introduction

Offshore wind energy plays a critical role in the worldwide transition to renewable energy sources, with floating wind turbines (FWTs) rendering a key technology to harness wind energy in open waters. FWTs offer access to high-wind regions located in deeper seas, enabling the tapping of stronger, more consistent wind resources, which help scale up offshore wind energy production while also reducing visual and environmental impacts near coastal areas. However, in these offshore areas, FWTs operate under harsh and complex environmental conditions, including strong winds, high wave loading, corrosion, continuous cyclic loading, and fluctuating temperatures. These factors pose significant challenges to the safe and efficient operation of these structures, increasing the risk of structural degradation and damage to critical components such as rotating parts, foundations, and mooring lines, which could affect the stability of FWTs. Over time, this highly risky situation could result in disastrous outcomes or possibly the asset’s complete loss [1]. Thus, it is evident that the detection of early stage damage in FWTs is vital for timely interventions that prevent damage from propagating into total failure. Early stage damage in FWTs refers to minor structural defects or degradation that have not yet significantly impacted the structure’s performance or safety but could potentially worsen uncontrollably over time and might also severely affect neighboring components.

Moreover, in offshore environments, where accessibility is limited, and repairs by maintenance teams are costly, demanding, and dangerous, remote and automated SHM provides the capability for predictive maintenance, helping to reduce unplanned downtime and extend the operating lifespan of such structures. Overall, a well-designed SHM system on FWTs may enhance safety, ensure proper operation, and enable predictive maintenance, with the latter two being essential for maximizing energy production while minimizing costs.

**Figure 1:**
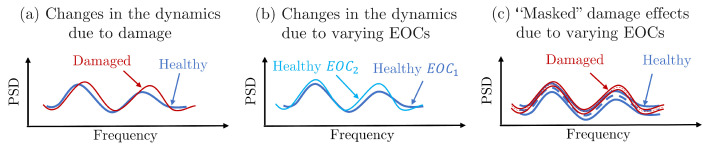
Schematic representation of the challenging damage diagnosis problem under varying EOCs: (**a**) damaged vs. healthy FWT under constant EOCs, (**b**) healthy FWT dynamics under two different EOCs, and (**c**) “masked” damage effects on the dynamics by those due to varying EOCs.

Among the various SHM techniques, vibration-based SHM has gained significant attention for its effectiveness in monitoring the dynamic behavior of a wide variety of structures. This technology is highly applicable, cost-effective, and capable of providing continuous, real-time monitoring. With the availability of high-quality, affordable sensors, large structural areas can be monitored with minimal equipment, making vibration-based SHM both practical and efficient. The core principle is that damages (e.g., cracks and joint loosening) induce changes in the structural dynamics by altering stiffness, mass distribution, and/or damping properties, which in turn affect the measurable vibration response of the structure [2,3]. The main challenge arises from the complex and varying environmental and operating conditions (EOCs) that significantly affect the vibration signals used by an SHM system. Differentiating between signal changes caused by damage and those induced by varying EOCs is particularly challenging, especially for early stage damage, where the subtle effects of damage can be easily masked by these variations, as indicatively illustrated in Figure 1. The ability to accurately differentiate the effects of varying EOCs from those caused by damage is critical for reducing false alarms, thus improving the reliability of the SHM system. Robust diagnostic methods are needed to handle this problem, ensuring that the system can operate autonomously with minimal false positives [4].

Data-driven vibration-based SHM methods are among the most widely utilized and have demonstrated high efficiency in diagnosing various types of damages in onshore and fixed-bottom offshore WTs operating under varying EOCs. By employing signal processing techniques, appropriate time and/or frequency domain features are extracted from the obtained signals to form damage-sensitive feature vectors. These vectors are then used to train machine learning (ML) algorithms, such as decision trees, artificial neural networks (ANNs), k-nearest neighbors (k-NN), or support vector machines (SVM) [5,6,7]. Moreover, features such as the above may be further processed using dimensionality reduction techniques, such as principal component analysis (PCA), where components that exhibit variability under a structure’s constant health state are discarded, assuming they are associated with the varying EOCs, while the remaining components are used for damage diagnosis [8,9,10]. The above methods have been effectively applied in diagnosing various levels of blade cracks [6,7,8,9,10], added mass on the blades [5], blade erosion, and connection degradation [6,7] under varying temperatures, wind conditions, and rotational speeds. Nevertheless, the effective implementation of most of the above methods requires a large data volume for their training, obtained from multiple sensors under varying EOCs, as well as the tuning of a significant number of hyperparameters.

Alternatively, data-driven approaches that rely on stochastic (data-based) parametric models, such as autoregressive (AR) models [11,12], linear parameter varying autoregressive (LPV-AR) models, and functional series time-dependent autoregressive (FS-TAR) models [13], have been demonstrated to offer robust damage diagnosis in the tower and blades of onshore wind turbines under a variety of wind and temperature conditions. Within these approaches, damage diagnosis is based on proper statistical testing using the model parameters or residuals as features, while their assessment is performed either via numerical simulations [13] or with experiments in the case of blade crack diagnosis [11,12]. Another approach that is based on the identification of physics-motivated stochastic subspace models achieves the detection of various undesired conditions, such as mechanical looseness between the pile and the tower, fouling, scouring, and structural inclination in a lab-scale monopile wind turbine operating under varying external forces implemented through the stochastic excitation from an electromagnetic shaker [14]. Similarly, simulating varying wind speeds via a shaker producing white noise of different amplitudes, different levels of crack damage have been successfully identified in the jacket foundation of a lab-scale monopile WT using k-NN and SVM classifiers, as reported in [15].

In contrast to onshore and fixed-bottom WTs, diagnosing damages on FWTs presents more difficulties because of their floating installation, which introduces further uncertainty. Existing research on FWTs under varying EOCs primarily focuses on the SHM of mooring systems, with a greater emphasis on mooring lines. Recent studies have predominantly addressed the detection, identification, and quantification of stiffness degradation in such lines [16,17,18,19,20,21], the assessment of biofouling levels [22], and the evaluation of fatigue damage [23] in mooring lines utilizing data-driven approaches. These include neural networks [19,20], fuzzy logic [18], deep neural networks [22], and hybrid approaches integrating physics-based models in state space with a data-driven k-NN method [21]. Furthermore, data-driven methods using vector autoregressive (VAR) [20] or transmittance function autoregressive with exogenous input (TF-ARX) models [24] have also been explored.

Based on the above, the research gap in the damage diagnosis of floating wind turbines can be viewed in two main aspects. On the one hand, the methods currently employed have primarily been assessed through simulations with numerical models that approximate damage in the mooring lines via stiffness degradation. On the other hand, there is a significant gap in addressing the diagnosis of early stage damage in other critical FWT components, such as the tower, the floater, and the blades, particularly under varying environmental operating conditions (EOCs).

The *goal* of the present study is the experimental investigation and comparative assessment of vibration-based ML SHM methods that could be incorporated into an SHM system for robust diagnosis, achieving initial damage detection and type identification, and finally, severity characterization with emphasis on early stage damages. In summary, the contributions of the study include the following: (i) the investigation of all stages of the damage diagnosis problem in an FWT under varying EOCs, (ii) the investigation of early stage damages in critical FWT components, aside from the mooring systems, and (iii) the assessment of ML SHM methods through a comprehensive experimental procedure.

The methods’ performance and comparison are assessed through hundreds of experiments with a lab-scale FWT model, which rotates normally under varying wind speeds and directions in healthy and damaged states. The latter includes three distinct types of subtle, early stage damages, whose effects on the observed dynamics are almost fully masked by those induced by varying wind conditions. More specifically, two distinct blade cracks of limited length, two different small added masses on the blade edge simulating possible accumulation of ice, and connection degradation at the mounting of the main tower with the floater are the five damage scenarios which are investigated in the study. All employed SHM methods operate using vibration signals from a single accelerometer, and their performance is investigated using damage-sensitive feature vectors that represent the structural dynamics, taking into account the whole considered frequency bandwidth rather than just static features such as the signal’s peak, RMS, and so on. The feature vectors arise from data-driven, non-parametric, and parametric stochastic modeling of the FWT dynamics through Welch-based power spectral density (PSD) estimates and estimation of the model parameters from multiple autoregressive (AR) models, respectively. Based on these vectors, two versions of an unsupervised multiple model (MM) method [25], which has demonstrated excellent performance in FWT diagnosis [26], are initially used for damage detection. Once damage is detected, two versions of its supervised form and corresponding versions of a supervised k-NN-based method [27] and an SVM-based method [28] are employed in the same framework for robust damage type identification and severity characterization.

The damage detection results of the study are illustrated using scatter plots that display the methods’ similarity distance metrics, along with receiver operating characteristic (ROC) curves, which depict the true positive rate (TPR) versus the false positive rate (FPR) [29]. Furthermore, confusion matrices [30] are used for damage type identification and severity characterization results. Flawless performance is defined as achieving a TPR of 100% for an FPR of 0% for damage detection and 100% total accuracy (see more details in Section 5.3) for damage identification and severity characterization.

It is noted that preliminary results from this study have been presented in our conference paper [26], where the diagnosis is limited to damage detection and identification. Two additional damage scenarios (a second smaller blade crack and a smaller added mass) that lead to a higher number of experiments are also considered in this study for the examination of the methods’ diagnostic limits, as well as for damage severity characterization, which was not investigated at all in the previous study. Furthermore, an SVM classifier combined with a Bayesian optimization-based method is also included in the robust diagnosis framework of this study, while the investigation and comparison of all methods’ performance using two global dynamics feature vectors, the PSD and the AR model parameters, are insightful additions.

The paper’s remaining sections are arranged as follows: the precise problem statement is outlined in Section 2, and the experimental procedure is comprehensively described in Section 3. In Section 4, the ML-type methods for robust diagnosis are presented, while their assessment and comparison are included in Section 5. Finally, a discussion of the results is presented in Section 6, followed by the final conclusions in Section 7.

## 2. Precise Problem Statement

The method’s training and normal operation requirements and assumptions for damage diagnosis are addressed in this study through their three main stages: (i) damage detection, (ii) damage type identification, and (iii) damage severity characterization. Initially, let us assume that the FWT operates under (almost) constant varying wind conditions (speed and direction in this study) throughout each batch of measurements during data acquisition. Based on this, the detailed description of each diagnosis stage is as follows.

**Stage 1: Damage Detection**

The problem of vibration-based damage detection is treated in an unsupervised manner as follows:

Given a set of *n* random vibration signals, $y_{i}\left[t\right]$ with i=1,…,n and t=1,…,N, where *t* represents normalized discrete time with respect to the sampling period, and *N* is the signal length in samples, acquired from the FWT operating under healthy state and wind conditions in the range of interest, the method’s training is performed.

Determine whether the current state of the FWT is healthy or damaged using a new random vibration signal $y_{u}\left[t\right]$ from an unknown FWT health state and wind condition.

**Stage 2: Damage Type Identification**

Once damage is detected, the second stage of the damage diagnosis procedure includes damage type identification. This problem is addressed in a supervised manner as follows:

Given a set of *n* (not necessarily equal to those used in Stage 1) random vibration signals $y_{i,j}\left[t\right]$ with j=1,…,m indicating a different type of damage, the method’s training is performed. As in Stage 1, this dataset includes measurements under the considered wind conditions.

Determine the type of detected damage using the random vibration signal $y_{u}\left[t\right]$ (subscript “*u*” indicates unknown), which is confirmed in Stage 1 to have originated from a damaged FWT health state.

**Stage 3: Damage Severity Characterization**

Once the damage type has been identified in the previous diagnosis stage, the objective of this stage is to characterize the severity of the damage in a supervised manner as follows:

Given a set of *n* (not necessarily equal to those used in the previous stages) random vibration signals $y_{i,j,z}\left[t\right]$ with z=1,…,l, indicating the different levels of severity investigated for each considered damage type, the method’s training is performed. As in the previous stages, these measurements are conducted in the considered range of wind conditions.

Determine the severity of the damage using the random vibration signal $y_{u}\left[t\right]$, which, based on Stage 2, is already known to correspond to specific damage of the considered types.

## 3. The Experimental Procedure

### 3.1. The Floating Wind Turbine (FWT) Lab-Scale Model

**Figure 2:**
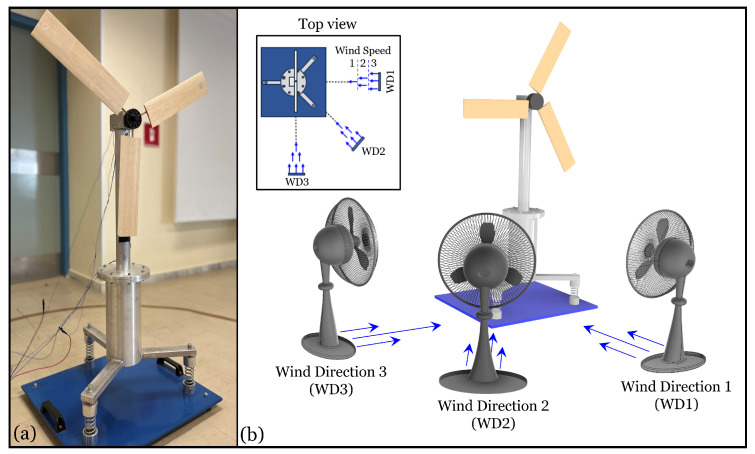
(**a**) Photo of the lab-scale FWT model and (**b**) the FWT under the considered wind directions (WD1, WD2, and WD3) and speeds (WS1, WS2, and WS3).

The lab-scale FWT model is shown in Figure 2a. Conceptualized and designed at the University of Patras and constructed by the company “Alphamach.gr”, it comprises several integral components: a central tower (450 mm height and 120 mm diameter), a rotor (56 mm diameter), blades (330×76 mm), a tension leg platform or “floater” (280 mm height and 126 mm diameter), a base plate (560×500×12 mm) supporting the entire structure, and three springs. The structure is predominantly constructed from aluminum, with exceptions being the iron base and springs and wooden blades. The tower houses the rotor, which is affixed to the blades and is secured at its lower section to the platform via bolted connections in a 10 mm thick flange. The total height of the structure, including the base and blades, is 1170 mm. The base plate and springs simulate the FWT’s motion, replicating the constraints from its sea-bottom mounting through typical mooring lines. The FWT model normally rotates under nine distinct operating conditions derived from three different wind speeds (WS1, WS2, and WS3) and three wind directions (WD1, WD2, and WD3). These operational conditions are attained by adjusting a fan’s rotational velocity and its direction relative to the FWT model, as depicted in Figure 2b.

### 3.2. Early Stage Damage Scenarios

**Figure 3:**
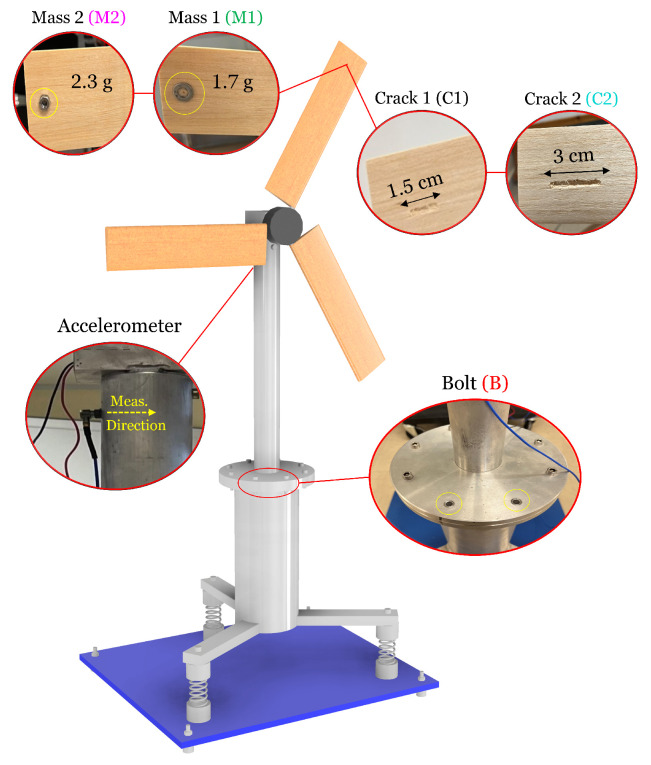
The lab-scale FWT model, the accelerometer position, and the considered damage scenarios.

Five different damage scenarios are investigated in this study, derived from three different types of early stage damage. These include one scenario of connection degradation between the tower and floater implemented by removing two of the eight mounting bolts, two scenarios of blade cracks, and two scenarios of added mass simulating potential ice accumulation. The last four scenarios are separately implemented on a single blade, with the two blade crack scenarios (C1 and C2) being implemented last due to their irreversibility. All damage scenarios, along with their abbreviations, are depicted in Figure 3 and described in more detail below:

1. Connection degradation between the tower and floater designated as ‘*B*’;
2. Added Mass 1 (m = 1.7 g), designated as ‘M1’;
3. Added Mass 2 (m = 2.3 g), designated as ‘M2’;
4. Blade Crack 1 (L = 1.5 cm, 4% of the blade’s overall length), designated as ‘C1’;
5. Blade Crack 2 (L = 3 cm, 8% of the blade’s overall length), designated as ‘C2’.

### 3.3. Vibration Signals

All vibration signals are acquired through the use of a single uniaxial lightweight accelerometer placed on the tower’s upper part, as shown in Figure 3, with a sampling frequency of $f_{s}=1024$ Hz (see details in Table 1). The data acquisition procedure is initially performed for the healthy FWT model under the minimum wind speed (WS1) and direction WD1. Ten vibration signals, each consisting of 30,720 samples (or 30 s long), are collected. After increasing the wind speed to WS2, another set of 10 signals is collected. This procedure is repeated for the maximum wind speed, WS3, as well. The same measurements are performed for wind directions WD2 and WD3, resulting in a total of 90 vibration signals for the healthy FWT. For each early stage damage scenario, the same procedure is used. Thus, 90 vibration signals are collected per FWT health state, leading to 540 vibration signals in total (Table 1) for training and assessing the ML SHM methods. The employed data acquisition system consists of a Compact-DAQ Ethernet chassis unit (cDAQ-9188) with eight slot ports from National Instruments (Austin, TX, USA), a data acquisition card (NIC Series 9230, three-channel, 51.2 kS/s for each channel) from National Instruments (Austin, TX, USA) that functions as the input module for vibrational measurements, and one piezoelectric accelerometer: Model 352C65 from PCB, 0.5 g, frequency range 1.0–10,000 Hz, sensitivity 1 mV/m/s^2^.

**Figure 4:**
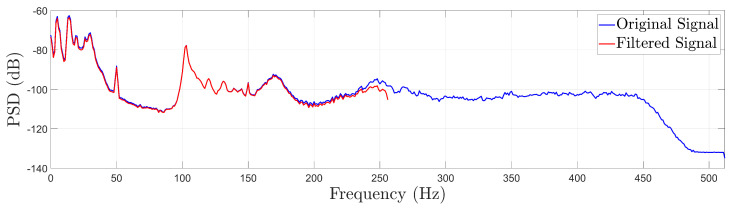
Indicative Welch—based PSD estimate using a vibration signal from the FWT in a healthy state before and after signal filtering and resampling at a frequency of $f_{s}=512$ Hz.

Following data acquisition, a 4th-order low-pass Chebyshev Type I filter with a cut-off frequency of 256 Hz and a ripple of 0.5 dB is applied to all vibration signals (*MATLAB R2023b function:* filtfilt.m). This signal pre-processing was chosen based on the fact that the frequency content of the signals above 256 Hz is not information-rich, as shown in the Welch-based PSD estimate ([31] p. 186) presented in Figure 4: Welch method parameters: Hamming window of 512 samples length, 90% overlap, frequency resolution δf=1 Hz. Then, all signals are resampled to the new sample frequency of $f_{s}=512$ Hz, as shown in Figure 4.

#### Effects of Varying Wind Condition and Early Stage Damages on Vibration Signals

In this subsection, the effects of the considered wind conditions, including both variations in speed and wind direction, as well as those due to the various early stage damage scenarios on the vibration signals, are explored. In particular, these effects are examined in the frequency domain using Welch-based PSD estimates obtained using a Hamming window of 512 samples in length and overlap of 90%, thus leading to a frequency resolution of δf=1 Hz.

**Figure 5:**
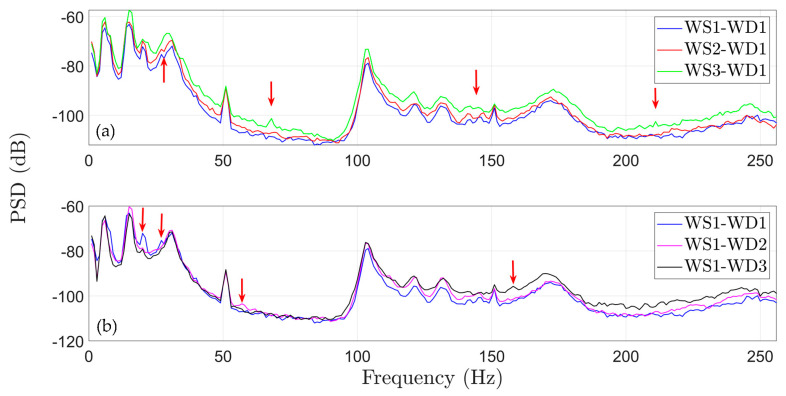
Indicative Welch—based PSD estimates using vibration signals from healthy FWT operating: (**a**) under all considered wind speeds (WS) for constant wind direction WD1 and (**b**) under all considered wind directions for constant wind speed WS1. The red arrows indicate significant changes in frequencies due to varying EOCs.

**Figure 6:**
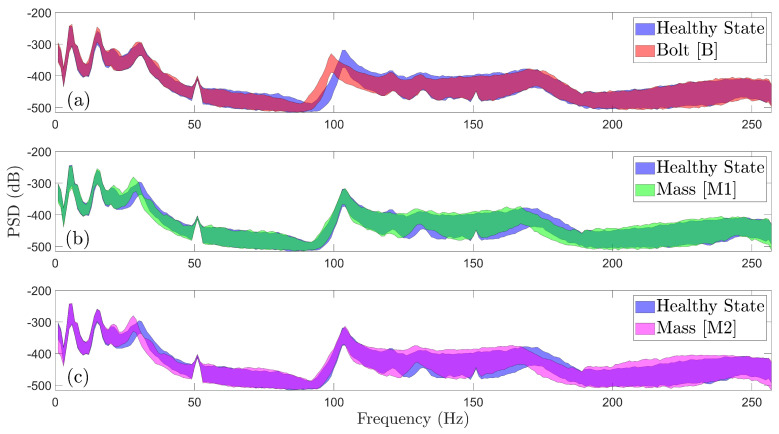
Welch—based PSD envelope estimates under all considered wind speeds and directions of the healthy and damaged FWT (90 signals per health state): (**a**) healthy vs. scenario B, (**b**) healthy vs. scenario M1, and (**c**) healthy vs. scenario M2.

**Figure 7:**
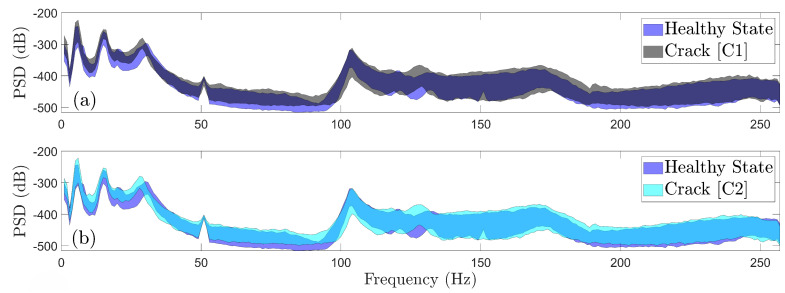
Welch—based PSD envelope estimates under all considered wind speeds and directions of the healthy and damaged FWT (90 signals per health state): (**a**) healthy vs. scenario C1 and (**b**) healthy vs. scenario C2.

Figure 5a demonstrates the effect of varying wind speed under a constant wind direction (WD1) on the PSD, utilizing three representative signals from the structure’s healthy state during normal rotation. On the other hand, Figure 5b illustrates the effects of the varying wind direction on the FWT dynamics while maintaining constant wind speed (WS1). The red arrows on the PSD graphs indicate some of the most prominent spectral variations due to varying EOCs. This is also confirmed through the PSD envelope of the healthy state in Figure 6, including all considered (varying) EOCs (see Table 1) in any of the three subplots. This envelope is the result of the accumulation of all distinct PSDs per different EOCs derived from 90 vibration signals. Similar envelopes are additionally provided for the considered damaged states of the FWT, as shown in Figure 6 and Figure 7.

Moreover, Table A1, Table A2 and Table A3 in Appendix A include the natural frequencies and damping ratios of the healthy FWT, as they were obtained from the AR models corresponding to the different wind conditions. Based on these, the variability of the structural dynamics with respect to the changing EOCs is also evident.

In these figures, the healthy state is depicted in blue, while red, green, magenta, black, and teal and corresponds to the *B*, M1, M2, C1, and C2 damage scenarios, respectively. Substantial overlap between the PSD envelopes of the healthy and damaged states is observed throughout most of the frequency range. This overlap arises from changes in the FWT dynamics due to varying wind conditions, which significantly affect the vibration signals and dominate the spectral characteristics, thus rendering accurate damage detection highly challenging.

**Figure 8:**
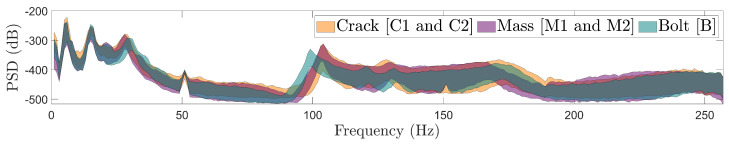
Effects of different damage types on the dynamics through Welch—based PSD envelope estimates for damage scenario B (90 signals), for scenarios C1 and C2 (180 signals), and for scenarios M1 and M2 (180 signals).

Additionally, the complexity of the damage type identification problem is demonstrated through the PSD envelopes depicted in Figure 8. These envelopes are derived from 90 vibration signals corresponding to damage scenario B and 180 vibration signals from the mass addition (M1 and M2) and cracks (C1 and C2) scenarios, including all considered wind speeds and directions. Evidently, there is a pronounced overlap between the PSD envelopes of various damage scenarios throughout most of the frequency range, indicating a challenging damage type identification problem.

**Figure 9:**
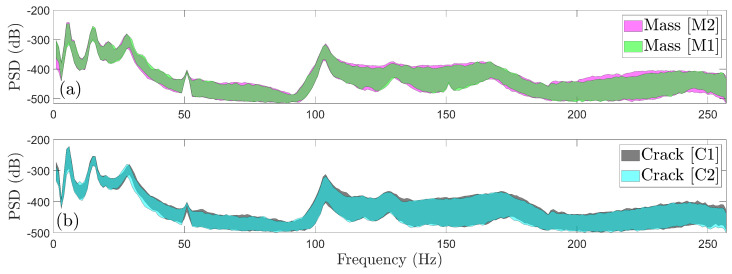
Effects of different damage severity levels for the same damage type through Welch—based PSD envelope estimates (90 signals per damage severity): (**a**) scenario M1 vs. scenario M2 and (**b**) scenario C1 vs. scenario C2.

Finally, Figure 9 illustrates the PSD envelopes derived from 90 vibration signals corresponding to the same damage type but different damage severity. The PSD envelopes for different severity levels are nearly indistinguishable for both (Figure 9a) mass and(Figure 9b) crack damage types. This similarity further highlights the significant difficulty in accurately characterizing damage severity under varying wind conditions.

## 4. Machine Learning Methods for Robust Damage Diagnosis

Each ML method employed for robust damage diagnosis belongs to the data-driven class and operates in two phases per stage of diagnosis (see Section 2). The first phase, typically called the *baseline phase*, includes the training of the method using vibration signals from known EOCs and the health status of the structure, while the second, the *inspection phase*, is performed continuously in real-time or periodically depending on the needs during the FWT operation. Furthermore, the performance of each ML method is assessed at each diagnosis stage using two different damage-sensitive feature vectors representing the structural dynamics within the entire considered frequency bandwidth. One feature vector includes Welch-based PSD estimates, and the other the parameters of autoregressive (AR) models obtained through standard identification procedures. In particular, PSD estimates are obtained using the Welch method (*MATLAB function:* pwelch.m), while each AR model is obtained based on a typical Least Squares estimator [31] (pp. 81–83) (*MATLAB function:* arx.m). AR model order selection is based on the Bayesian information criterion (BIC) and the residual-to-signal sum of squares ratio (RSS/SSS%) [31] (pp. 505–507). Its validation is achieved through typical model residual whiteness examination [31] (pp. 512–513); see Section 5.2. In this context, *damage detection (diagnosis stage 1)* is pursued unsupervised using the U-MM-AR and U-MM-PSD methods, while *damage type identification (diagnosis stage 2)* and *severity characterization (diagnosis stage 3)* are conducted supervised via the S-MM-AR, S-MM-PSD, k-NN-AR, k-NN-PSD, SVM-AR, and SVM-PSD methods; ‘U’ and ‘S’ designate unsupervised and supervised methods, respectively.

### 4.1. Stage 1: Multiple Model (MM)-Based Robust Damage Detection

*Baseline (training) phase:* In this phase, the MM representation [25] of the healthy FWT dynamics, Mo, is constructed via multiple individual models, obtained either parametrically (for U-MM-AR) or non-parametrically (for U-MM-PSD) using the available vibration signals (also see Stage 1 in Section 2). Each model is referred to as $M_{o,i}$, with subscript “o” designating a healthy structural state and i=1,…,n determining the dimensionality of $M_{o}$. The estimation of each model is achieved using a single vibration signal from the healthy FWT under a specific wind condition in the considered range (also see Section 2).

*Inspection (detection) phase:* In this phase, damage detection is performed under normal operating conditions. A fresh vibration signal is obtained with the FWT being under an unknown health state. Based on this, a new model, say $M_{u}$, is estimated, and damage detection is based on determining whether or not the new model $M_{u}$ belongs to the MM representation $M_{o}$. In the positive case, the FWT is declared “healthy” or “damaged”.

The decision-making mechanism is based on a similarity distance metric *D* between the new model $M_{u}$ and $M_{o}$. This is currently defined as the minimum distance between $M_{u}$ and all elements of $M_{o}$:

(1) $$D:=\min_{i}d(M_{o,i},M_{u}),\;\mathrm{for}i=1,\ldots ,n,$$

with $d(M_{o,i},M_{u})$ designating a statistical distance between the two individual models $M_{o,i}$ and $M_{u}$. For the U-MM-PSD method, the Euclidean distance is employed:

(2) $$d\left(M_{o,i},M_{u}\right)=\sqrt{{\left({\hat{S}}_{o,i}-{\hat{S}}_{u}\right)}^{T}\left({\hat{S}}_{o,i}-{\hat{S}}_{u}\right)},$$

with ${\hat{S}}_{o,i}$ and ${\hat{S}}_{u}$ designating estimates of the Welch-based PSD magnitude corresponding to $M_{o,i}$ and $M_{u}$ models, respectively (a hat over a symbol designates estimate). On the other hand, for the U-MM-AR method, the Mahalanobis distance is employed:

(3) $$d\left(M_{o,i},M_{u}\right)=\sqrt{{\left({\hat{\theta }}_{o,i}-{\hat{\theta }}_{u}\right)}^{T}{\left({\hat{P}}_{o,i}\right)}^{-1}\left({\hat{\theta }}_{o,i}-{\hat{\theta }}_{u}\right)},$$

with ${\hat{\theta }}_{o,i}$ and ${\hat{\theta }}_{u}$ designating the estimated AR parameter vectors associated with $M_{o,i}$ and $M_{u}$ models, respectively, while ${\hat{P}}_{o,i}$ designates the estimated covariance matrix of ${\hat{\theta }}_{o,i}$, as obtained through the the Cramèr-Rao bound ([31] p. 218). Damage detection is then declared if and only if *D* is greater than a user-specified threshold $L_{lim}$:

(4) $$\begin{array}{c} \;D\leq L_{lim}\to HealthyFWT,\\ \;Else\;\to DamagedFWT. \end{array}$$

### 4.2. Stage 2: ML Methods for Robust Damage Type Identification

#### 4.2.1. MM-Based Damage Identification

*Baseline (training) phase:* In this phase, a single MM representation of the FWT dynamics under each of the *m* considered damage types is constructed. Consequently, *m* MM representations designated as $M_{j}$ (j=1,2,…,m) are obtained, with each $M_{j}$ corresponding to a specific damage type. Each $M_{j}$ is constructed via multiple individual models, obtained either parametrically (for S-MM-AR) or non-parametrically (for S-MM-PSD), using the available vibration signals (see stage 2 in Section 2). Each model is referred to as $M_{j,i}$, where i=1,…,n determines the dimensionality of $M_{j}$. The estimation of each model $M_{j,i}$ is achieved using a single vibration signal obtained from the FWT operating under the jth damage type and a specific wind condition in the considered range.

*Inspection (identification) phase:* This phase involves damage type identification under normal operation of the FWT. Once the new vibration signal is determined (Stage 1) to originate from a damaged health state of the FWT, the model $M_{u}$ estimated during stage 1 is also utilized in this phase. Damage type identification is then performed by determining which of the previous (training) phase representations $M_{j}$ the model $M_{u}$ belongs. This is achieved, as in damage detection, using a similarity distance metric $D_{type}$ to evaluate the proximity of $M_{u}$ to each $M_{j}$. Specifically, $D_{type}$ is defined as the minimum distance between $M_{u}$ and all individual elements within each $M_{j}$:

(5) $$D_{type}:=\min_{i}d(M_{j,i},M_{u}),\;\mathrm{for}j=1,\ldots ,m\;\mathrm{and}\;i=1,\ldots ,n,$$

with $d(M_{j,i},M_{u})$ designating a statistical distance between the two individual models, which is the Euclidean distance for the S-MM-PSD method and the Mahalanobis for the S-MM-AR method, similar to damage detection (see Section 4.1).

#### 4.2.2. k-NN-Based Damage Identification

*Baseline (training) phase*: In this phase, a single k-NN class representing the dynamics of the FWT for each of the *m* considered damage types is developed. Thus, *m* classes are determined with their respective class labels and are designated as $T_{j}$, with j=1,2,…,m. Each class comprises a set of individual feature vectors obtained either parametrically (for k-NN-AR) or non-parametrically (for k-NN-PSD). These individual feature vectors are labeled as $T_{j,i}$, where i=1,…,n, with *n* determining the $T_{j}$ dimensionality. Each $T_{j,i}$ is estimated using a single vibration signal obtained from the FWT operating under the jth damage type and a specific wind condition within the considered range (see Stage 2 in Section 2).

*Inspection (identification) phase:* The feature vector (PSD or AR parameter vector) denoted as $T_{u}$ obtained from model $M_{u}$ is employed for damage type identification. This is achieved by classifying $T_{u}$ into one of the $T_{j}$ k-NN classes from the training phase. For this purpose, the *K* nearest neighbors of $T_{u}$ are selected based on a distance metric between $T_{u}$ and each $T_{j,i}$ (j=1,2,…,m, i=1,2,…,n). Finally, the class (damage type) of each of the *K* neighbors is counted, and the class with the maximum participation (i.e., the most “votes”) is selected as the class to which $T_{u}$ belongs.

It is noted that for both k-NN versions, the tuning procedure of the hyperparameters, including the number of nearest neighbors, the distance metric, and the distance weight, is conducted based on Bayesian optimization [32] (pp. 2951–2959) (see Section 5.3).

#### 4.2.3. SVM-Based Damage Identification

SVM classification is primarily a binary classification technique that must be adapted to address multi-class problems. Identifying the type of detected damage using SVM belongs to the context of multi-class classification. Therefore, the common one vs. all technique is employed [33].

*Baseline (training) phase*: In this phase, *m* classifiers, one for each damage type, are developed. For each classifier, *n* signals derived across the considered range of wind conditions, along with a set of corresponding parametric (for SVM-AR) or non-parametric (for SVM-PSD) feature vectors $\left\{\alpha \right\}=\left\{\alpha_{1},\ldots ,\alpha_{m\cdot n}\right\}$, are utilized.

The SVM algorithm [34] (pp. 131–146) aims to construct an optimal separating hyperplane (or other hypersurface) that effectively separates two classes, which is expressed by the following function:

(6) $$\begin{array}{c} D\left(\alpha \right)=w^{T}\varphi \left(\alpha \right)+b, \end{array}$$

where the weight vector w (perpendicular to the hyperplane) and the coefficient *b* are used to define the position and orientation of the separating hyperplane, while *T* denotes the transpose operation. The choice of any φ(α)≠α transforms the hyperplane into a hypersurface in the original coordinate system determined from set α. The goal of the training phase is the determination of the decision function D(α) (Equation (6)) that describes the optimal separating hyperplane by ensuring that the classification error in the baseline set is minimized while still maximizing the distance between the hyperplane and the closest to hyperplane baseline feature vectors of each class in {α}.

In order to quantify the classification error in the baseline set, a non-negative slack variable $\xi_{i}$ (i=1,…,m·n) for each $\alpha_{i}$ is introduced. Indeed, each $\xi_{i}$ represents the distance between the hyperplane and the feature vector $\alpha_{i}$ when lying on the wrong side of the margin. A feature vector $\alpha_{i}$ is misclassified if $\xi_{i}\geq 1$.

Assuming that $D(\alpha_{i})\geq 1$ for $\alpha_{i}$ coming from class A, and $D(\alpha_{i})\leq -1$ for $\alpha_{i}$ coming from class B, the distance between the hyperplane and the nearest to the hyperplane element in {α} from each class is defined as follows:

(7) $$\begin{array}{c} d\left(\alpha ,w,b\right)=\frac{1}{{∥w∥}_{2}}, \end{array}$$

with ${∥\cdot ∥}_{2}$ designating the $l_{2}$ norm, and the geometrical margin between the two structural states is given by the quantity $\frac{2}{{∥w∥}_{2}}$. It is noted that the concept of the margin is fundamental to the SVM framework, as it is a measure of its generalization capability.

D(α) may be determined as the solution of the following convex quadratic programming problem:

(8) $$\begin{array}{c} \min_{w,b,\xi }\frac{1}{2}{∥w∥}_{2}+C\sum_{i=1}^{m\cdot n}\xi_{i},\\ Y_{i}\left(w^{T}\varphi \left(\alpha_{i}\right)+b\right)\geq 1-\xi_{i},\;\xi_{i}\geq 0,\;i=1,\ldots ,m\cdot n. \end{array}$$

The first term in the criterion ensures margin maximization between two classes, and the second provides an upper bound for the classification error in the baseline set. *C* is the error penalty that controls the balance between training error and margin maximization, $Y_{i}=1$ if $\alpha_{i}$ is obtained from the first class A, and $Y_{i}=-1$ if it is obtained from class B [34]. In order to simplify this optimization problem and eliminate the constraints, the method of Lagrange multipliers [34] (pp. 131–146) is used. Thus, the optimization problem of Equation (8) is transformed into the following dual quadratic form:

(9) $$\begin{array}{c} \max_{r}\sum_{i=1}^{m\cdot n}r_{i}-\frac{1}{2}\sum_{i,j=1}^{m\cdot n}r_{i}r_{j}Y_{i}Y_{j}\left(\varphi^{T}\left(\alpha_{i}\right)\cdot \varphi \left(\alpha_{j}\right)\right),\\ \mathrm{subject}\;\mathrm{to}:\;\sum_{i=1}^{m\cdot n}r_{i}Y_{i}=0,\;0\leq r_{i}\leq C,\;i=1,\ldots ,m\cdot n, \end{array}$$

where the coefficients $r_{i}$ are the Lagrange multipliers, and the constant *b* is calculated as referred to in [34] (pp. 134–135). If there is a kernel function such that $K\left(\alpha_{i},\alpha_{j}\right)=\varphi^{T}\left(\alpha_{i}\right)\cdot \varphi \left(\alpha_{j}\right)$, the explicit calculation of φ(α) turns out to be unnecessary [28]. The most popular kernel functions are the linear $K\left(\alpha_{0,i},\alpha_{0,j}\right)=\alpha_{0,i}^{T}\cdot \alpha_{0,j}$, the polynomial $K\left(\alpha_{0,i},\alpha_{0,j}\right)={\left(\gamma \alpha_{0,i}^{T}\cdot \alpha_{0,j}+1\right)}^{d}$, and the Gaussian $K\left(\alpha_{0,i},\alpha_{0,j}\right)=\exp\left(-\gamma ∥\alpha_{0,i}-\alpha_{0,j}{∥}^{2}\right)$. The tuning of hyperparameters γ and *d* is based on Bayesian optimization.

Thus, by solving the dual optimization problem (Equation (9)), one obtains Lagrange multipliers $r_{i}$’s (i=1,…,m·n). The decision function associated with the optimal hyperplane turns out to be the following:

(10) $$\begin{array}{c} D\left(\alpha \right)=\sum_{i=1}^{m\cdot n}r_{i}Y_{i}K\left(\alpha_{i},\alpha \right)+b. \end{array}$$

Training is performed for *m* binary classifiers, where each classifier derives a decision boundary D(α) based on the procedure outlined above; thus, *m* hyperplanes D(α) are constructed. In each classifier, class A represents a specific damage type, while class B includes all the remaining types. This approach enables the differentiation between the various types of detected damage. For both SVM-AR and SVM-PSD, the tuning procedure of the hyperparameters, including the kernel function, kernel scale, and box constraint, is conducted based on the Bayesian Optimization approach [32] (pp. 2951–2959) (see Section 5.3).

*Inspection (identification) phase*: The feature vector $\alpha_{u}$ corresponding to model $M_{u}$ is herein utilized for damage type identification. The D(α) values are calculated for each classifier based on Equation (10), and the (unknown) feature vector $\alpha_{u}$ is assigned to class A of the classifier with the highest positive D(α).

### 4.3. Stage 3: ML Methods for Robust Damage Severity Characterization

#### 4.3.1. MM-Based Damage Severity Characterization

*Baseline (training) phase:* In this phase, a single MM representation of the FWT dynamics for each of the *l* considered damage severity levels under varying wind conditions is constructed for each of the *m* damage types. This leads to l×m MM representations, designated as $M_{j}^{z}$ (j=1,…,m and z=1,…,l), with each $M_{j}^{z}$ corresponding to a damage severity level *z* of damage type *j*. As previously mentioned, the feature vectors of multiple individual parametric (S-MM-AR) or non-parametric (S-MM-PSD) models compose each $M_{j}^{z}$ representation. The individual models are labeled as $M_{j,i}^{z}$, where i=1,…,n indicate the number of signals used for model identification (see Stage 3 in Section 2) and *n* defines the $M_{j}^{z}$ dimensionality. Each model $M_{j,i}^{z}$ is estimated using a single vibration signal obtained from the FWT operating in the jth damaged state with a zth damage severity level under a specific wind condition within the considered range.

*Inspection (severity characterization) phase:* The model $M_{u}$ is utilized, and severity characterization is performed by determining which of the training phase representations, $M_{j}^{z}$, belongs. It is noted that *j*, corresponding to the damage type, is available from diagnosis stage 2. Thus, damage severity characterization is achieved using a distance metric, $D_{mag}$, which evaluates the proximity of $M_{u}$ to each $M_{j}^{z}$. Specifically, $D_{sev}$ is defined as the minimum distance between $M_{u}$ and all individual elements within each $M_{j}^{z}$:

(11) $$D_{sev}:=\min_{i}d(M_{j,i}^{z},M_{u})\;\mathrm{for}z=1,\ldots ,l\;\mathrm{and}\;i=1,\ldots ,n,$$

with $d(M_{j,i}^{z},M_{u})$ designating the employed statistical distance between the two individual models. In particular, the Euclidean distance is selected for the S-MM-PSD method and the Mahalanobis for the S-MM-AR method (see Section 4.1).

#### 4.3.2. k-NN-Based Damage Severity Characterization

*Baseline (training) phase*: In this phase, a single k-NN class for the representation of the FWT dynamics for each of the *l* considered damage severity levels under varying wind conditions is constructed for each of the *m* damage types. Thus, m×l classes are defined along with their class labels, designated as $T_{j}^{z}$, with j=1,2,…,m and z=1,…,l. Each class comprises a set of individual feature vectors, either parametric (for k-NN-AR) or non-parametric (for k-NN-PSD). The individual feature vectors are labeled as $T_{j,i}^{z}$ with i=1,…,n and *n*, defining the $T_{j}$ dimensionality as above for the MM method. Each $T_{j,i}^{z}$ is estimated using a single vibration signal obtained from the FWT operating in the jth damaged state with a zth damage severity level under a specific wind condition within the considered range. Bayesian optimization [32] (pp. 2951–2959) is utilized for the method’s hyperparameter tuning (see Section 5.3).

*Inspection (severity characterization) phase:* The model $M_{u}$ is utilized, and the corresponding feature vector, denoted as $T_{u}$, is employed for damage severity characterization. This is achieved by classifying the feature vector $T_{u}$ into one of the $T_{j}^{z}$ k-NN classes from the training phase. For this purpose, the *K* nearest neighbors of $T_{u}$ are selected based on a statistical distance between $T_{u}$ and each $T_{j,i}^{z}$ (i=1,2,…,n, z=1,…,l, *j*: identified from diagnosis stage 2). Eventually, the class (damage severity) of each of the *K* neighbors is counted, and the class with the highest involvement is selected as the class to which $T_{u}$ belongs.

#### 4.3.3. SVM-Based Damage Severity Characterization

*Baseline (training) phase*: In this phase, the construction of m·l individual classifiers for each of the *l* considered damage severity levels under varying wind conditions is constructed for each of the *m* damage types. Again, the goal here is the determination of the decision function D(α) of Equation (6), describing the optimal separating hyperplane. Following a similar procedure as described in Section 4.2.3, the decision function associated with the optimal hyperplane within the damage type, which has already been identified (stage 2), finally turns out to be the following:

(12) $$\begin{array}{c} D_{j}\left(\alpha \right)=\sum_{i=1}^{l\cdot n}r_{i}Y_{i}K\left(\alpha_{i},\alpha \right)+b\;\mathrm{for}j=1,\ldots ,m. \end{array}$$

Thus, m·l binary classifiers corresponding to m·l decision boundary/optimal hyperplanes $D_{j}\left(\alpha \right)$ for j=1,⋯,m are developed, with each consisting of a set of individual parametric (SVM-AR) or non-parametric (SVM-PSD) feature vectors.

In this case of damage severity characterization, for each classifier of a specific damage type *m*, class A represents an individual severity level, while class B includes all the remaining levels. This technique allows for clear differentiation between the different severity levels of the same damage type. The hyperparameter tuning of kernel function, kernel scale, and box constraint is performed using Bayesian optimization [32] (pp. 2951–2959) (see Section 5.3).

*Inspection (severity characterization) phase*: The feature vector $\alpha_{u}$ of model $M_{u}$, corresponding to a specific damage type in the FWT as determined during the diagnosis Stages 1 and 2, is utilized, and the $D_{j}\left(\alpha \right)$ values (*j* is known from stage 2) are calculated for each classifier based on Equation (12). The unknown signal is categorized into class A of the classifier with the maximum $D_{j}\left(\alpha \right)$.

## 5. Experimental Results

### 5.1. Performance Assessment Procedure

The performance assessment of the methods across all three stages (stage 1: damage detection, stage 2: damage type identification, and stage 3: damage severity characterization) is performed using an iterative “rotation” approach similar to the k-fold cross-validation ([30] p. 33), in order to separate the available data into training and testing sets. This strategy reduces potential bias introduced by specific vibration signals used in training, ensuring statistically robust assessment and fair comparison of the methods. For stage 1, in the first rotation, a random (*MATLAB function:* randperm.m) subset of vibration signals from the FWT in a healthy state is selected to train the damage detection methods, while the remaining signals are reserved for the inspection phase. In the second rotation, a different random subset of healthy signals is utilized for the training phase, while the remaining signals are used in the inspection phase. This procedure is repeated until all available signals have been included in the training phase at least once. A similar procedure is applied for stages 2 and 3, with the difference that, within each rotation, an equal number of vibration signals for the baseline and inspection phase are always randomly selected from each damage type in stage 2 and each damage severity level in stage 3.

Twenty rotations are performed in this study for the methods’ performance assessment and comparison, resulting in 900 inspection test cases for the healthy state and 1800 test cases for each of the considered damage scenarios (*B*, M1, M2, C1, and C2). This results in a total of 9900 inspection test cases for the *damage detection* problem (see Table 2). For *damage type identification*, 900 inspection test cases are used for scenario *B*, and 1800 test cases for each scenario of the *M* and *C* damage types, yielding a total of 4500 inspection test cases (see Table 3). Lastly, for *damage severity characterization*, 900 inspection test cases are used for damage severity levels associated with *M* (M1 and M2) and *C* (C1 and C2) damage types (see Table 4).

In addition, it should be noted that for both stages 2 and 3, in the training phase of the k-NN and SVM-based methods for the hyperparameters tuning procedure via Bayesian optimization, a typical k-fold cross-validation technique has also been utilized. This is used only within the first rotation (see Table 3 and Table 4) of the training phase, where Bayesian optimization has been conducted for a random subset of the available signals. The hyperparameters are selected and kept constant within the rest of the rotations. More specifically, in this approach, the set of training signals obtained from the first rotation is split into k equal portions. The evaluation process involves using one portion as the validating set, while the remaining k − 1 portions serve as the training set. This procedure is repeated k times, with each portion taking a turn as the validating set, while the others are used for training. In this study, a five-fold cross-validation method is employed within the first rotation of damage type identification and damage severity characterization.

### 5.2. Damage Detection Results

*Baseline (training) phase*: In this phase, n=45 vibration signals are used per rotation (see Table 2) to construct the MM representation $M_{o}$ of the healthy FWT dynamics under the considered wind conditions. For the U-MM-AR method, the MM representation $M_{o}$ consists of n=45 autoregressive models of order $n_{a}=80$, denoted as AR (80). On the other hand, $M_{o}$ for the U-MM-PSD method consists of n=45 Welch-based PSD estimates with all estimation details for both methods presented in Table 5.

**Figure 10:**
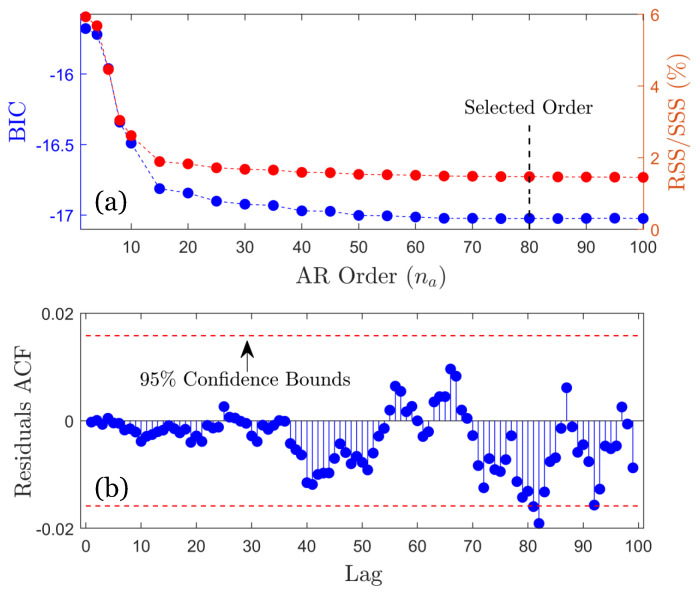
(**a**) Model order selection via the BIC criterion (blue line) and corresponding residual sum of squares over the signal sum of squares percentage (RSS/SSS%) (red line) with the selected order indicated at $n_{a}=80$; (**b**) model validation via the residual auto correlation function (ACF) at a 95% confidence level.

AR model order selection based on the BIC and RSS/SSS% is shown in Figure 10a, using an indicative vibration signal from the healthy FWT, while model validation is shown in Figure 10b through the residuals autocorrelation function (ACF) within the 95% confidence bounds.

*Inspection (detection) phase*: Each vibration signal from 9900 test cases of the inspection phase (see Table 2) is treated as originating from an unknown health state. For each vibration signal, a new model $M_{u}$ is estimated. This is an AR (80) for the U-MM-AR method, while a Welch-based PSD estimate of the signal using the same estimation details with the baseline phase for the U-MM-PSD method.

**Figure 11:**
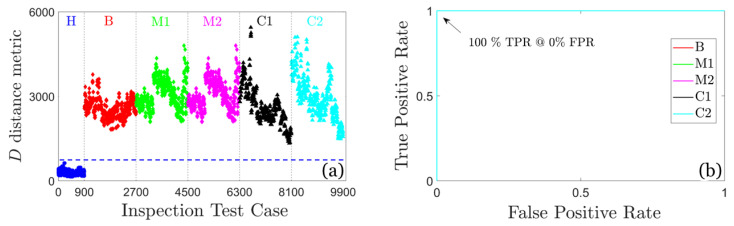
Damage detection results based on the U-MM-AR method: (**a**) plot of the distance metric *D* and (**b**) corresponding ROC curves (overlapped due to perfect performance); 900 inspection test cases for the healthy FWT and 1800 per damage scenario, 9900 in total. The blue dashed line indicates the user selected threshold.

The detection results for the U-MM-AR method are illustrated in Figure 11. Evidently, the values of the distance metric *D* corresponding to the FWT’s healthy state are completely distinguishable from those of early stage damages, demonstrating the method’s flawless damage detection performance. This is further confirmed by the ROC curves, which indicate a perfect detection rate (100% TPR) with no false alarms (0%, FPR) for all considered damage scenarios.

**Figure 12:**
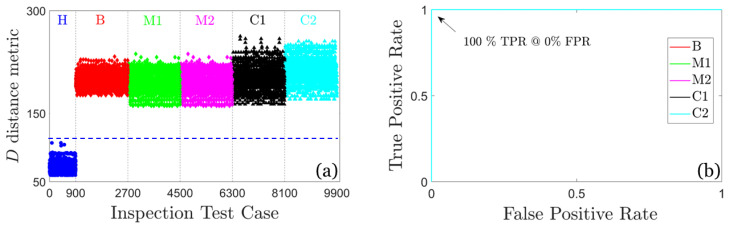
Damage detection results based on the U-MM-PSD method: (**a**) plot of the distance metric *D* and (**b**) corresponding ROC curves (overlapped due to perfect performance); 900 inspection test cases for the healthy FWT and 1800 per damage scenario, 9900 in total. The blue dashed line indicates the user selected threshold.

Similarly, Figure 12 illustrates the values of the distance metric *D* and the ROC curves for the U-MM-PSD method. It is again evident that the detection performance is perfect (100% TPR and 0% FPR) for all considered damage scenarios.

### 5.3. Damage Type Identification Results

*Baseline (training) phase*: In this phase, n=45 vibration signals from the bolt (B) damage type and n=90 signals from each of the remaining damage types are used per rotation (see Table 3) to construct MM representations $M_{j}$ (j=1,2,3). Each $M_{j}$ captures the FWT dynamics for a specific damage type under the considered wind conditions, as described in Section 4.2.1. For the S-MM-AR method, each $M_{j}$ representation consists of 225 AR (80) models, while for the S-MM-PSD method, it consists of 225 Welch-based PSD estimates. All details on the estimation procedures are provided in Table 6.

Using the same set of vibration signals, a single k-NN class $T_{j}$ (j=1,2,3) is created to represent the FWT dynamics for each damage type over the considered wind conditions, as described in Section 4.2.2. As for MM-based methods, for the k-NN-AR method, each $T_{j}$ includes 225 AR (80) parameter vectors, while for the k-NN-PSD method, it includes 225 Welch-based PSD estimates.

Finally, the same set of vibration signals is also used to train m=3 binary SVM classifiers, one for each damage type, based on the procedure described in Section 4.2.3. Again, the training of the SVM-AR method is performed utilizing 225 AR (80) parameter vectors, while 225 Welch-based PSD estimates are used for the SVM-PSD method. For both SVM and k-NN-based methods, the tuning procedure of the hyperparameters has been conducted based on the Bayesian optimization approach [32] (pp. 2951–2959). This optimization algorithm constructs a probabilistic model of the classification error and leverages it to make informed decisions about which hyperparameter values are more likely to minimize the error. The Bayesian optimization process incorporates an acquisition function to determine the subsequent set of hyperparameter values for evaluation. The fundamental principle underlying Bayesian optimization is the utilization of all available information from previous classification error evaluations rather than relying solely on local gradient approximations. The details about the objective function, the acquisition function and the search intervals of the hyperparameters are shown in Table 7, while the final hyperparameter values are provided in Table 6.

*Inspection (identification) Phase*: Each vibration signal from the 4500 test cases in the inspection phase (see Table 3), which has been previously (Stage 1) identified to originate from a damaged health state, is in this stage used for damage type identification. Thus, each model $M_{u}$, estimated (see Table 6) during stage 1, is now used for damage type identification through the methods outlined in Section 4.2.1, Section 4.2.2 and Section 4.2.3.

**Figure 13:**
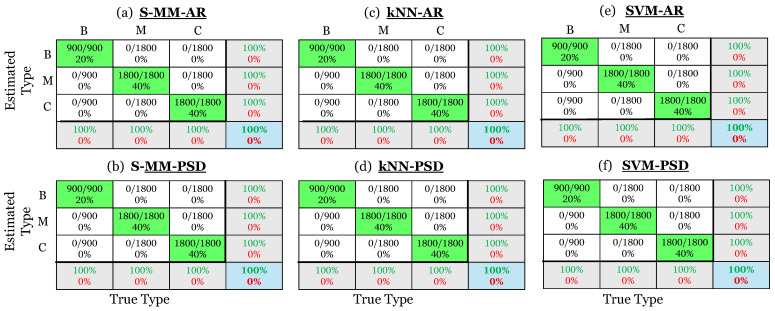
Damage type identification results via confusion matrices. (**a**) The S-MM-AR method, (**b**) the S-MM-PSD method, (**c**) the k-NN-AR method, (**d**) the k-NN-PSD method, (**e**) the SVM-AR method, and (**f**) the SVM-PSD method. Correct identification is indicated by green color and misidentification by red (4500 inspection test cases in total).

The damage type identification results are presented through confusion matrices in Figure 13. Each column of a confusion matrix in the upper left 3 × 3 sub-matrix corresponds to the actual type of early stage damage, with each row representing the predicted type. The *i*,*j*-th cell indicates the number of times the actual *i*-th damage type was predicted as the *j*-th damage type, presented as a ratio in relation to the total number of actual inspection test cases. Along the diagonal, the true damage type is correctly identified, while in the off-diagonal parts, it is incorrectly identified. The percentages of correctly (in green) and incorrectly (in red) identified cases out of all predictions made for each specific type are shown in the column on the far right of the matrix. The bottom row shows the percentages of correctly (in green) and incorrectly (in red) identified cases out of all actual cases of that type. Finally, the cell at the bottom and right indicate the overall correct identification rate (in green) and false identification rate (in red) across all three types. In other words, this cell presents the total accuracy of the method, while the rightmost column and the bottom row indicate the typical precision and recall ratios, respectively, for each type [35]. As is evident, both versions of all investigated methods demonstrate excellent performance in damage type identification, achieving an overall correct identification rate of 100%.

### 5.4. Damage Severity Characterization Results

*Baseline (training) phase*: In this phase, n=45 vibration signals from each damage severity level of the added mass and the blade crack scenarios are used per rotation (see details in Table 4) to construct the MM representations $M_{j}^{z}$ (j=1,2 and z=1,2). Each $M_{j}^{z}$ captures the FWT dynamics for a specific damage type and severity level for the considered wind conditions, as described in Section 4.3.1. For the S-MM-AR method, the $M_{j}^{z}$ consists of 180 AR (80) models, while 90 Welch-based PSD estimates are employed for the S-MM-PSD method. All estimation details are provided in Table 8.

Using the same set of vibration signals, a single k-NN class $T_{j}^{z}$ (j=1,2 and z=1,2) is created for the representation of the FWT dynamics for each considered damage severity and damage type, as described in Section 4.3.2. As previously mentioned, each $T_{j}^{z}$ includes 180 AR (80) parameter vectors for the k-NN-AR method and 180 Welch-based PSD estimates for the k-NN-PSD method.

Finally, the same set of vibration signals is used to train z=2 binary SVM classifiers for each damage type, one for each damage severity level, based on the procedure described in Section 4.3.3. Thus, for the training of the SVM-AR method, 180 AR (80) parameter vectors are utilized, while for the SVM-PSD method, 180 Welch-based PSD estimates are used. The tuning procedure of the hyperparameters of SVM and k-NN-based methods is conducted based on Bayesian optimization [32] (pp. 2951–2959), as described in Section 5.3 and detailed in Table 7. The final hyperparameter values of the methods are provided in Table 8.

*Inspection (severity characterization) phase*: Each vibration signal from the 1800 test cases in the inspection phase (see Table 4), which was previously identified to originate from a specific type of damage, is used for damage severity characterization in this diagnosis stage. Thus, each model $M_{u}$ is employed for damage severity characterization using the methods outlined in Section 4.3.1, Section 4.3.2 and Section 4.3.3. Similar to Section 5.3, in the first version of each method, $M_{u}$ corresponds to an AR (80) model, whereas in the second version, it corresponds to a Welch-based PSD estimate. All details of each method are summarized in Table 8.

**Figure 14:**
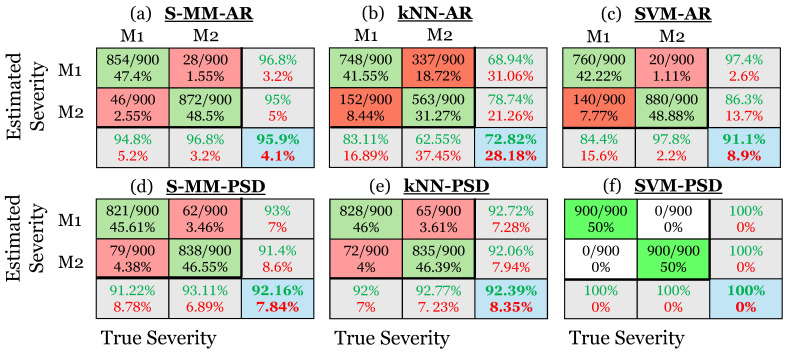
Damage severity characterization results via confusion matrices for the added mass damage scenario. (**a**) The S-MM-AR-based method, (**b**) the k-NN-AR-based method, (**c**) the SVM-AR-based method, (**d**) the S-MM-PSD-based method, (**e**) the k-NN-PSD-based method, and (**f**) the SVM-PSD-based method. Correct characterization is indicated by green color and mischarachterization by red (1800 inspection test cases in total).

The damage severity characterization results are presented using confusion matrices, similar to those in Section 5.3, with the difference that each column of a confusion matrix in the upper left 2 × 2 sub-matrix corresponds to the actual severity of the investigated damage, with each row representing the predicted one. Correspondingly, the cell at the bottom right indicates the total correct characterization rate (accuracy) and false characterization rate across the two levels of severity. Specifically, Figure 14 illustrates the confusion matrices corresponding to the results of all investigated methods for the added mass (M1 and M2) severity characterization problem. Both versions of the MM-based method demonstrate adequate performance, achieving correct severity characterization rates of 95.9% and 92.16%, respectively. From the k-NN-based methods, the non-parametric k-NN-PSD has superior performance over its parametric counterpart, achieving a correct characterization rate of 92.39%, while the k-NN-AR method achieves a poor rate of 72.82%. Finally, the SVM-PSD method achieves perfect damage severity characterization performance with a 100% correct estimation rate, while its parametric counterpart, the SVM-AR method, achieves a rate of 91.1%. More accuracy measures for damage severity characterization are presented in Appendix B.

**Figure 15:**
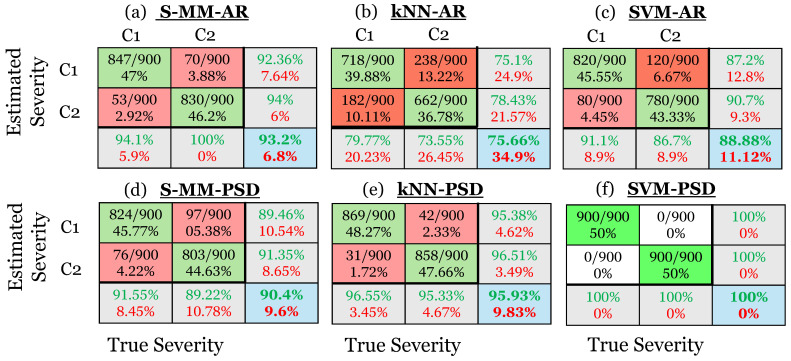
Damage severity characterization results via confusion matrices for the blade crack damage scenario. (**a**) The S-MM-AR-based method, (**b**) the k-NN-AR-based method, (**c**) the SVM-AR-based method, (**d**) the S-MM-PSD-based method, (**e**) the k-NN-PSD-based method, and (**f**) the SVM-PSD-based method. Correct characterization is indicated by green color and mischaracterization by red (1800 inspection test cases in total).

Moreover, Figure 15 presents the confusion matrices for all investigated methods in the blade crack (C1 and C2) severity characterization problem. Both versions of the MM-based method demonstrate adequate performance, achieving severity characterization rates of 93.2% and 90.4% based on the S-MM-AR and S-MM-PSD methods, respectively. On the other hand, the k-NN-AR method has poor performance, achieving 75.66% accuracy in characterizing the blade crack severity, whereas the k-NN-PSD method achieves a very good rate of 95.93%. Finally, the SVM-PSD method again demonstrates perfect performance with 100% accuracy in characterizing the blade crack severity, while its SVM-AR counterpart exhibits inferior performance with an accuracy rate of 88.88%.

**Figure 16:**
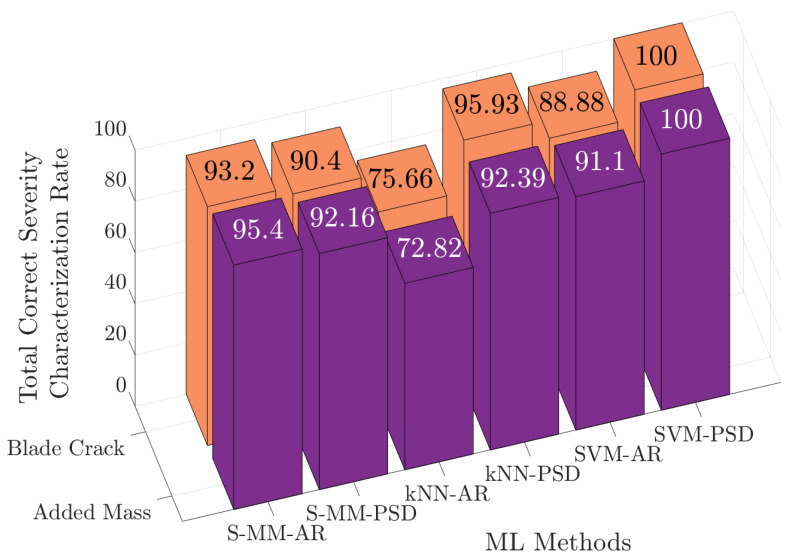
Summary of the correct severity characterization percentages for damage scenarios with added mass (M1,M2) and blade crack (C1,C2) based on all considered methods.

For a direct comparison of all methods, the overall results of damaged severity characterization for the added mass (M1 and M2) and blade crack (C1 and C2) damage scenarios are presented as bar graphs in Figure 16. The best severity characterization performance for both damage types is achieved by the SVM-PSD method, while the lowest is achieved by the k-NN-AR method.

## 6. Discussion

Based on the above results, it is evident that the investigated vibration-based ML methods for robust SHM may lead to a highly effective SHM system in an FWT operating under a number of uncertainty sources. The methods demonstrate perfect performance in both damage detection and damage type identification of the early stage, while even in the delicate stage of damage severity characterization, the SVM-PSD method achieves 100% correct detection with zero false alarms. Regarding the methods’ complexity and thus their practical value for real-time use, it is noted that MM-based methods, which are used for damage detection, operate in a completely unsupervised manner without the need for measurements from the damaged structure, while they require a limited number of vibration signals from a single sensor on the healthy FWT lab-scale model for its training. More specifically, only 8.33% of the total 10800 signals from the healthy FWT are used in the training of the baseline phase for damage detection, with the remaining signals employed exclusively in the inspection phase.

It is noted that for the stages of damage type identification and severity characterization, the same number of signals has been utilized for the training of all methods, ensuring consistency in the methods’ assessment and comparison. The signals were utilized equally in the baseline phase (50%) and inspection phase (50%), examining the methods’ performance, leading to flawless identification of all damage types based on either of the investigated methods. This may be attributed to damage-sensitive feature vectors representing the structural dynamics within the entire measurements’ frequency bandwidth. The results from damage severity characterization are perfect based on the SVM-PSD method followed by the k-NN-PSD and S-MM-AR methods, with the remaining methods achieving correct characterization rates of around 90% and higher except from the k-NN-AR method, which is inadequate. It is worth mentioning that the training of such classification methods is based on a sample of damage scenarios, as it is impossible to have all damage scenarios available, and based on these, they perform the classification of an unknown damage scenario to the closest known from the baseline phase [36,37] with, as expected, reduced accuracy, particularly in damage characterization.

Furthermore, it should be stressed that both k-NN and SVM-based methods are considerably more complex and computationally demanding than MM-based methods due to their dependency on extensive hyperparameter tuning. Since the same features are employed across the three investigated methods, it is important to note that certain hyperparameters are common to all methods regarding each version. Specifically, the methods’ version utilizing PSD estimates requires the determination of the overlap, window length, and window type, while the other, employing AR parameter vectors, requires only the order of the AR models to be determined. MM-based methods are inherently less complex, as they do not require further hyperparameters to be determined, making their setup and implementation in an SHM system simpler and faster. On the other hand, Bayesian optimization has been utilized to fine-tune three extra hyperparameters in the baseline phase of SVM-based and k-NN-based methods. Particularly, the kernel function, kernel scale, and box constraint level were determined for SVM-based methods, while the number of nearest neighbors, the distance metric, and the distance weight were assessed for k-NN-based methods. This optimization process necessitates a thorough exploration of the hyperparameter space, involving multiple iterations of model performance evaluation, which significantly increases computational cost and time requirements.

Finally, it should be noted that interpretability and transparency are very important for the user of an SHM system. Compared to complex deep neural network architectures, the employed methods offer advantages in both interpretability and transparency. This is particularly evident in MM-based methods, which incorporate a simple decision-making mechanism and require minimal hyperparameter tuning. Similarly, k-NN-based methods provide clear insights into why specific predictions are made thanks to their transparent procedure. However, reduced interpretability and transparency, due to increased complexity, are observed in SVMs utilizing non-linear kernel functions, where decision boundaries become more involved, and data are mapped and transformed into high-dimensional spaces, as also confirmed in [38].

## 7. Conclusions

The experimental investigation and comparative assessment of established ML vibration-based SHM methods for robust diagnosis of early stage damage in floating wind turbines (FWTs) under varying environmental and operating conditions (EOCs) have been presented. Two versions of three advanced ML methods were employed in a proper framework for robust diagnosis: a multiple model (MM) method, a k-nearest neighbors (k-NN) method, and a support vector machines (SVM) method. Each version incorporates two damage-sensitive feature vectors that represent the structural dynamics within the entire frequency bandwidth of measurements, one that includes Welch-based PSD estimates and another with autoregressive (AR) model parameters.

The performance of the methods has been rigorously evaluated through hundreds of experiments, demonstrating their effectiveness in the detection, type identification, and severity characterization of very early stage damages, whose effects on the vibration signals are almost completely masked by those induced by varying wind conditions. The MM-based method, which is characterized by fewer hyperparameters and the simplest implementation, achieved flawless damage detection and type identification while achieving correct damage severity characterization for over 90% of the considered cases using either of its two versions. The k-NN-based method also achieved flawless damage type identification, but only its k-NN-PSD version may reach acceptable (>92%) damage severity characterization. Finally, the SVM-based methods demonstrated the best performance, as it achieved perfect damage type identification based on either of its two versions, as well as flawless (100%) severity characterization through its SVM-PSD version with the SVM-AR method to be close to an average of 90%.

## Figures and Tables

**Table 1:** Experimental details on varying wind conditions, FWT health states, and vibration signals.

FWT Health State	Wind Speed (WS)	Wind Direction (WD)	No. of Exp. per WS/WD	Total No. of Exp.
Healthy (H)	{1, 2, 3}	{1, 2, 3}	10/10	90
Bolt (B)	-//-	-//-	-//-	-//-
Mass 1 (M1)	-//-	-//-	-//-	-//-
Mass 2 (M2)	-//-	-//-	-//-	-//-
Crack 1 (C1)	-//-	-//-	-//-	-//-
Crack 2 (C2)	-//-	-//-	-//-	-//-
**Original signals**:				
Sampling rate: $f_{s}=1024$ Hz; Length: N = 30,720 samples; Frequency bandwidth: [0–512] Hz				
**Pre-processed signals**:				
Sampling rate: $f_{s}=512$ Hz; Length: N = 15,360 samples; Frequency bandwidth: [0–256] Hz				
Pre-processing: Low-pass Chebyshev Type I filter and resampling				

**Table 2:** Damage detection: details on the performance assessment.

*Baseline (Training) Phase*						
**No. of Rotations**	**Healthy State**	**Bolt (B)**	**Mass 1 (M1)**	**Mass 2 (M2)**	**Crack 1 (C1)**	**Crack 2 (C2)**
1	45 ^*a*^	–	–	–	–	–
20	900	–	–	–	–	–
***Inspection (Detection) Phase***						
1	45 ^*a*^	90	90	90	90	90
20	900	1800	1800	1800	1800	1800

^*a*^ Five signals per wind condition. Different signals for each phase. In total, 45 training signals per rotation; 9900 inspection signals in total.

**Table 3:** Damage type identification: details on the performance assessment.

*Baseline (Training) Phase*			
**No. of Rotations**	**Bolt (B)**	**Mass (M1, M2)**	**Crack (C1, C2)**
1	45 ^*a*^	90 ^*b*^	90 ^*b*^
20	900	1800	1800
***Inspection (Identification) Phase***			
1	45 *^a^*	90 ^*b*^	90 ^*b*^
20	900	1800	1800

^*a*^ Five signals per wind condition. Different signals for each phase. ^*b*^ In total, 45 signals per damage type scenario (5 signals per wind condition); 225 training signals per rotation; 4500 inspection signals in total.

**Table 4:** Damage severity characterization of added mass and blade crack damage scenarios: details on the performance assessment.

*Baseline (Training) Phase*		
**No. of Rotations**	**Mass 1 (M1)**/**Crack 1 (C1)**	**Mass 2 (M2)**/**Crack 2 (C2)**
1	45 ^*a*^	45 ^*a*^
20	900	900
***Inspection (Severity Characterization) Phase***		
1	45	45
20	900	900

^*a*^ Five signals per wind condition. Different signals for each phase. In total, 90 training signals per rotation; 1800 inspection signals in total.

**Table 5:** Damage detection: details on the method’s training and inspection phases.

Method	Feature	Feature Vector Dimensionality	Distance Type
U-MM-AR	AR parameter vector	80	Mahalanobis
U-MM-PSD	PSD estimates	256	Euclidean
***Baseline (Training) Phase***			
AR model estimation via OLS ([31] p. 204), *MATLAB function:* ar.m			
Selected model: AR(80); BIC: −17.11; Samples Per Parameter (SPP): 192;			
Condition Number: $1.92\times 10^{5}$			
***Inspection (Detection) Phase***			
*U-MM-AR (U-MM-PSD)*: Detection based on the minimum Mahalanobis (Euclidean) distance			
User defined threshold: 600 (90)			

**Table 6:** Damage type identification: details on the method’s baseline and inspection phases.

Method	Feature	Feature Vector Dimensionality	Distance Type
S-MM-AR	AR parameter vector	80	Mahalanobis
k-NN-AR	-//-	-//-	Cosine
SVM-AR	-//-	-//-	-
S-MM-PSD	PSD estimates	256	Euclidean
k-NN-PSD	-//-	-//-	Cosine
SVM-PSD	-//-	-//-	-
***Baseline (Training) Phase***			
AR model estimation via OLS ([31] p. 204), *MATLAB function:* ar.m			
Selected model: AR(80); BIC: −17.11; Samples Per Parameter (SPP): 192;			
Condition Number: $1.92\times 10^{5}$			
***Inspection (Identification) Phase***			
*k-NN-AR:* Search Method: Exhaustive; No. of Nearest Neighbors: K=2; BreakTies: Nearest;			
Weight: Inverse			
*k-NN-PSD:* Search Method: Exhaustive; No. of Nearest Neighbors: K=3; BreakTies: Nearest;			
Weight: Equal (no weighting)			
*SVM-AR:* Kernel Function: Quadratic; Kernel scale: 1; Box Constrain level: 1.852;			
Multi-class coding: One vs. All			
*SVM-PSD:* Kernel Function: Gaussian; Kernel scale: 0.008; Box Constrain level: 22;			
Multi-class coding: One vs. All			

**Table 7:** Hyperparameter selection details.

ML Methods	Hyperparameters	Search Interval
	Kernel type	{Linear, Quadratic, Gaussian, Cubic}
SVM-based methods	Kernel scale	$\left[10^{-3},10^{3}\right]$
	Box constrain level	$\left[10^{-3},10^{3}\right]$
		{Euclidean, City block, Chebyshev,
	Distance Metric	Mahalanobis, Cosine, Correlation,
k-NN-based methods		Hamming, Jaccard, Minkowski}
	Distance Weight	{Equal, Inverse, Squared inverse}
	No. of Nearest Neighbors	[1, No. of training signals]
Bayesian Optimization details:		
Objective function: minimum classification error; Acquisition function: expected improvement (EI)		
Objective function evaluations: 30; Number of initial evaluation points: 4; Exploration ratio: 0.5		
k-fold cross-validation: k = 5		

**Table 8:** Damage severity characterization: details on the method’s baseline and inspection phases.

Method	Feature	Feature Vector Dimensionality	Distance Type
S-MM-AR	AR parameter vector	80	Mahalanobis
k-NN-AR	-//-	-//-	Minkowski (Euclidean) ^1^
SVM-AR	-//-	-//-	-
S-MM-PSD	PSD estimates	256	Euclidean
k-NN-PSD	-//-	-//-	Euclidean (Corelation) ^1^
SVM-PSD	-//-	-//-	-
***Baseline (Training) Phase***			
AR model estimation via OLS ([31] p. 204), *MATLAB function:* ar.m			
Selected model: AR(80); BIC: −17.11; Samples Per Parameter (SPP): 192;			
Condition Number: $1.92\times 10^{5}$			
***Inspection (Severity Characterization) Phase***			
^1^*k-NN-AR:* Search Method: Exhaustive (Exhaustive); No. of Nearest Neighbors: k=30(2);			
BreakTies: Nearest (Nearest); Weight: Squared Inverse (Inverse)			
^1^*k-NN-PSD:* Search Method: Exhaustive (Exhaustive); No. of Nearest Neighbors: K=1(1);			
BreakTies: Nearest (Nearest); Weight: Squared Inverse (Inverse)			
^1^*SVM-AR:* Kernel Function: Linear (Cubic); Kernel scale: 1(1);			
Box Constrain level: 247(0.715); Multi-class coding: One vs. All (One vs. All)			
^1^*SVM-PSD:* Kernel Function: Linear (Gaussian); Kernel scale: 1(951);			
Box Constrain level: 0.001(883); Multi-class coding: One vs. All (One vs. All)			

^1^ The values within the (parentheses) correspond to the blade crack scenario. The rest of the values correspond to the added mass scenario.

## Data Availability

Dataset available on request from the authors.

## Abbreviations

The following abbreviations are used in this manuscript:

AR	Autoregressive
BIC	Bayesian Information Criterion
EOCs	Environmental and Operating Conditions
FPR	False Positive Rate
FWT	Floating Wind Turbine
k-NN	k-Nearest Neighbors
ML	Machine Learning
MM	Multiple Model
OLS	Ordinary Least Squares
PCA	Principal Components Analysis
PSD	Power Spectral Density
ROC	Receiver Operating Characteristic
RSS	Residual Sum of Squares
SHM	Structural Health Monitoring
STS	Statistical Time Series
SPP	Samples Per Parameter
SVM	Support Vector Machines
TPR	True Positive Rate
WD	Wind Direction
WS	Wind Speed

## Appendix A

**Table A1:** Natural frequencies and damping ratios under all considered wind speeds for constant wind direction WD1.

Wind Direction Constant (WD1)					
**WS1**		**WS2**		**WS3**	
**Natural**	**Damping**	**Natural**	**Damping**	**Natural**	**Damping**
**Frequencies**	**Ratios**	**Frequencies**	**Ratios**	**Frequencies**	**Ratios**
6.5207	0.8891	6.1926	0.8623	7.9937	7.9937
11.4592	0.4048	11.4453	0.4108	11.7797	0.4078
18.2357	0.2662	18.2251	0.2793	18.7558	0.2665
30.6377	0.1674	30.6020	0.1802	30.5380	0.1805
49.4911	0.0343	49.7082	0.0415	49.8036	0.0454
105.5939	0.0502	105.0291	0.0451	104.9758	0.0462
118.3079	0.0223	117.9206	0.0250	117.3300	0.0249
135.0025	0.0310	138.9671	0.0182	134.1185	0.0525
155.9154	0.0105	156.5037	0.0147	155.9195	0.0156

Natural Frequencies in Hz.

**Table A2:** Natural frequencies and damping ratios under all considered wind speeds for constant wind direction WD2.

Wind Direction Constant (WD2)					
**WS1**		**WS2**		**WS3**	
**Natural**	**Damping**	**Natural**	**Damping**	**Natural**	**Damping**
**Frequencies**	**Ratios**	**Frequencies**	**Ratios**	**Frequencies**	**Ratios**
5.7398	0.8978	5.2527	0.8103	5.6493	0.8726
10.6856	0.3887	10.7696	0.3529	10.8213	0.3779
17.2765	0.3000	17.3295	0.2717	17.6490	0.2809
29.1436	0.1651	29.1447	0.1676	29.7297	0.1747
49.1931	0.0402	49.3667	0.0431	49.5073	0.0542
104.1938	0.0742	105.4680	0.0451	106.6280	0.0548
117.6449	0.0242	117.5488	0.0230	116.9707	0.0317
134.8372	0.0427	138.6149	0.0424	136.0300	0.0714
156.1472	0.0144	156.5427	0.0127	155.4205	0.0210

Natural Frequencies in Hz.

**Table A3:** Natural frequencies and damping ratios under all considered wind speeds for constant wind direction WD3.

Wind Direction Constant (WD3)					
**WS1**		**WS2**		**WS3**	
**Natural**	**Damping**	**Natural**	**Damping**	**Natural**	**Damping**
**Frequencies**	**Ratios**	**Frequencies**	**Ratios**	**Frequencies**	**Ratios**
4.8510	0.7857	4.9460	0.8195	4.8108	0.8444
10.6856	0.3887	10.5753	0.3082	10.4151	0.2824
17.4587	0.2467	17.3093	0.2549	17.4056	0.2429
29.1574	0.1474	29.0969	0.1456	28.9238	0.1494
49.1742	0.0302	49.1256	0.0345	48.9635	0.0474
103.6780	0.0505	103.6611	0.0432	103.4873	0.0410
116.4424	0.0246	116.1785	0.0230	115.4553	0.0265
138.7180	0.0250	136.2871	0.0361	135.0528	0.0321
153.7418	0.0180	154.5287	0.0127	153.8308	0.0301

Natural Frequencies in Hz.

## Appendix B

This appendix includes an additional statistical analysis, which is indicatively performed for damage severity characterization, including the determination of further accuracy measures (see Table A4 and Table A5) that prove the independence of runs within the rotation procedure. The values of the accuracy measures are presented for each considered method as obtained from the 20 rotations. For a better understanding of the tables and taking the S-MM-AR method as an example, the method achieves its maximum accuracy of 96.66% at one random rotation while its minimum of 85.55% at another rotation. The mean and median values for this method are 91.72% and 92.22%, respectively, indicating its consistent performance across rotations. The total accuracy of the method that corresponds to the correct severity characterization as obtained from all inspection test cases (from all rotations) reaches 92.16%. Based on this analysis, it is evident that each run may lead to a different accuracy value, ensuring statistically robust results, which are independent of specific vibration signals that may be used for the methods’ training in a certain rotation. The total accuracy values, also shown in the confusion matrices’ rightmost and lowest cell, provide each method’s overall performance in damage severity characterization.

**Table A4:** Statistical accuracy measures for severity characterization of added mass scenarios (20 Rotations).

Accuracy (%)	S-MM-AR	S-MM-PSD	kNN-AR	kNN-PSD	SVM-AR	SVM-PSD
Max	96.66	96.66	80.00	97.77	95.55	100.00
Min	85.55	86.66	65.55	86.66	82.22	100.00
Mean	91.72	92.16	72.83	92.38	91.00	100.00
Median	92.22	92.77	73.33	92.77	91.66	100.00
Total	92.16	95.90	72.82	92.39	91.10	100.00

**Table A5:** Statistical accuracy measures for severity characterization of blade crack scenarios (20 Rotations).

Accuracy (%)	S-MM-AR	S-MM-PSD	kNN-AR	kNN-PSD	SVM-AR	SVM-PSD
Max	95.55	96.66	78.88	97.77	92.22	100.00
Min	87.78	85.55	64.44	87.77	81.11	100.00
Mean	93.16	92.16	76.66	95.94	88.88	100.00
Median	92.77	92.77	77.77	96.67	90.00	100.00
Total	93.20	90.40	75.66	95.93	88.88	100.00
